# Supplementary material for: Hormetic Response of Photosystem II Function Induced by Nontoxic Calcium Hydroxide Nanoparticles
Source: Int J Mol Sci. 2024 Jul 30;25(15):8350. doi: 10.3390/ijms25158350 (PMC11312163; doi:10.3390/ijms25158350)
Supplement: Supplementary file 1 [file ijms-25-08350-s001.zip › ijms-3122682-supplementary.pdf]

International Journal of *Molecular Sciences*

MDPI

# Hormetic Response of Photosystem II Function Induced by Nontoxic Calcium Hydroxide Nanoparticles

**Panagiota Tryfon [1], Ilektra Sperdouli [2,*], Julietta Moustaka [3], Ioannis-Dimosthenis S. Adamakis [4], Kleoniki Giannousi [1], Catherine Dendrinou-Samara [1] and Michael Moustakas [5]**

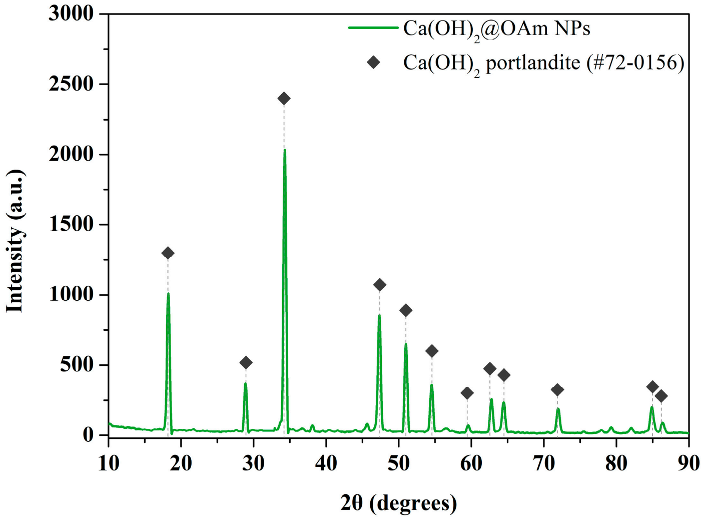


**Figure S1.** X-ray diffraction (XRD) of the synthesized $Ca(OH)_2$@OAm NPs.

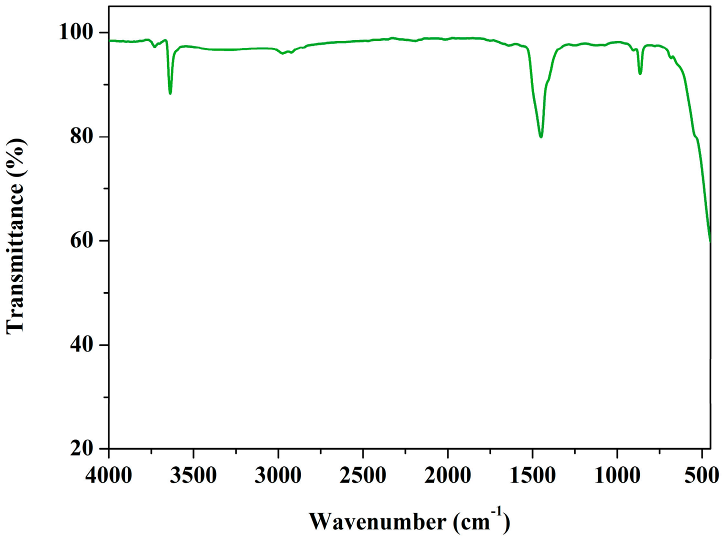


**Figure S2.** Fourier-transform infrared (FT-IR) spectrum of $Ca(OH)_2$@OAm NPs (4000 – 450 $cm^{-1}$).

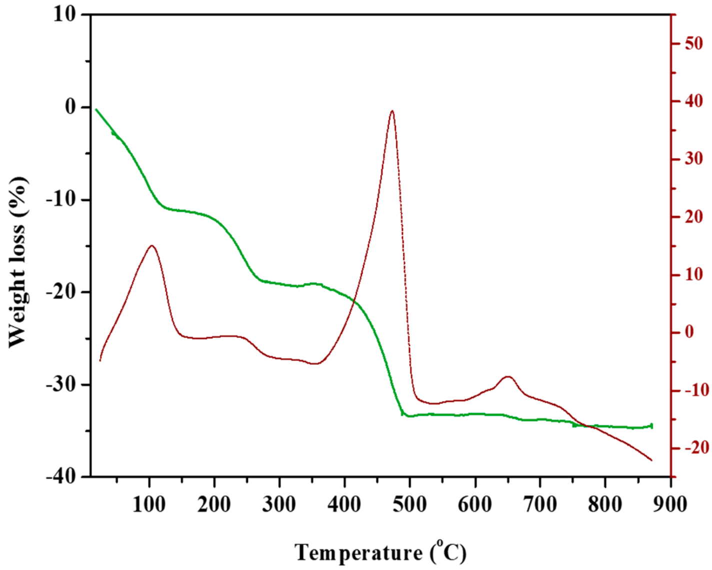


**Figure S3.** Thermogravimetric analysis (TGA) and derivative thermogravimetric (DTG) of the NPs.

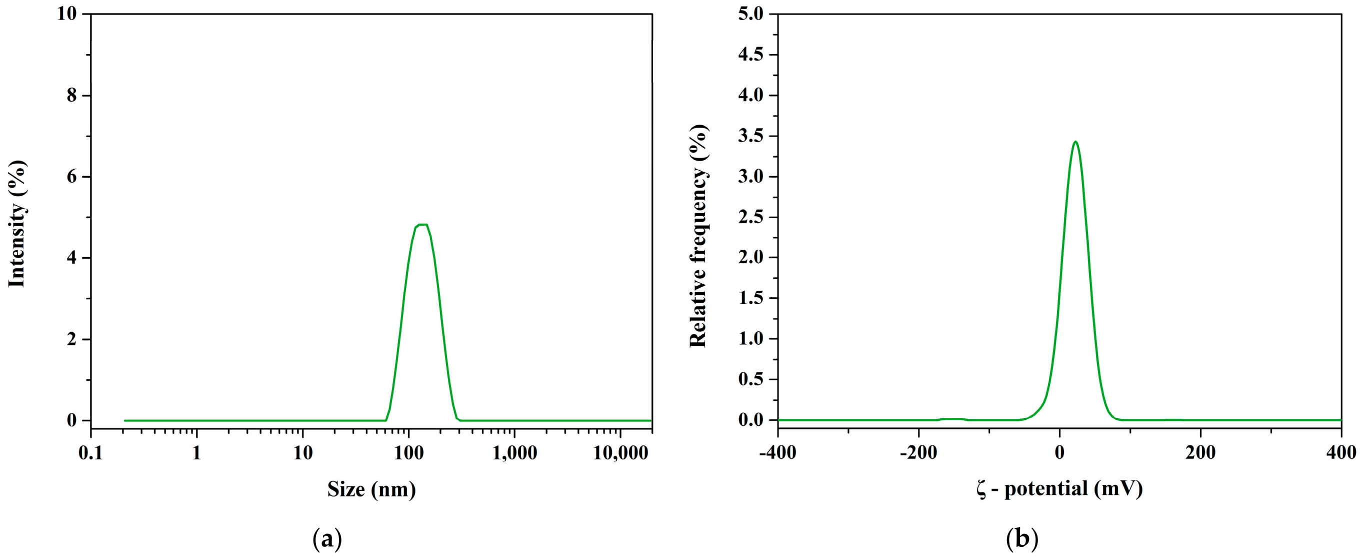


**Figure S4.** Hydrodynamic diameter distribution **(a)** and ζ-potential **(b)** of $Ca(OH)_2$@OAm NPs.

**Table S1. Definitions of the chlorophyll fluorescence parameters used in the experiments**

| Parameter | Definition | Calculation |
|---|---|---|
| F*v*/F*m* | Maximum efficiency of PSII photo-chemistry | (F*m* − F*o*)/F*m* |
| $\Phi_{PSII}$ | Effective quantum yield of PSII photo-chemistry | (F*m*′ − F*s*)/F*m*′ |
| $\Phi_{NPQ}$ | Quantum yield of regulated non-photochemical energy loss in PSII | F*s*/F*m*′ − F*s*/F*m* |
| $\Phi_{NO}$ | Quantum yield of nonregulated energy | F*s*/F*m* |

| | | |
|---|---|---|
| | loss in PSII | |
| F*v*′/F*m*′ | Efficiency of open PSII reaction centers | (F*m*′ − F*o*′)/F*m*′ |
| F*v*/F*o* | Efficiency of the oxygen evolving complex (OEC) on the donor side of PSII | (F*m* − F*o*)/F*o* |
| ETR | Electron transport rate | $\Phi_{PSII}$ × PAR × c × abs, where PAR is the photosynthetically active radiation, c is 0.5, and abs is the total light absorption of the leaf taken as 0.84 |
| q*p* | Photochemical quenching, representing the redox state of quinone A ($Q_A$), or in other words the fraction of PSII reaction centers in open state based on the "puddle" model for the photosynthetic unit | (F*m*′ − F*s*)/(F*m*′ − F*o*′) |
| NPQ | Non-photochemical quenching reflecting the dissipation of excitation energy as heat | (F*m* − F*m*′)/F*m*′ |
| EXC | Excess excitation energy | (F*v*/F*m* − $\Phi_{PSII}$)/F*v*/F*m* |
